# Facile Synthesis of MoP and Its Composite Structure with Ru as an Efficient Electrocatalyst for Hydrogen Evolution Reaction in Both Acidic and Alkaline Conditions

**DOI:** 10.3390/ma18051184

**Published:** 2025-03-06

**Authors:** Pinyun Ren, Rui Wang, Yujie Yang, Tianyu Wang, Yilun Hong, Yi Zheng, Qianying Zheng, Xianpei Ren, Zhili Jia

**Affiliations:** 1 School of Photoelectric Engineering, Changzhou Institute of Technology, Changzhou 213032, China; 2 Material Corrosion and Protection Key Laboratory of Sichuan Province, Artificial Intelligence Key Laboratory of Sichuan Province, College of Physics and Electronic Engineering, Sichuan University of Science and Engineering, Zigong 643000, China; 3 Center for Advanced Measurement Science, National Institute of Metrology, Beijing 100029, China

**Keywords:** phosphorization, Ru/MoP composite, electrocatalysis, hydrogen evolution reaction

## Abstract

Developing low-cost electrocatalysts for efficient hydrogen evolution in both acidic and alkaline conditions is crucial for water-electrolytic hydrogen applications. Herein, MoP was synthesized via a simple, low-cost, and green phosphorization route. More importantly, the Ru/MoP composite prepared using the as-synthesized MoP as a reactant, which exhibited excellent catalytic activity for the hydrogen evolution reaction. It showed lower overpotentials of 108 and 55 mV at 10 mA·cm^−2^ in acidic and alkaline solutions, respectively, which are superior to those of bare Ru and pristine MoP as well as comparable or even better than those of previously reported excellent Ru- or MoP-based catalysts. In addition, it also demonstrated small Tafel slopes of 52.6 mV dec^−1^ and 67.9 mV dec^−1^ in acidic and alkaline solutions, respectively, along with long-term stability. This work provides an effective and feasible route to design high-efficient MoP-based electrocatalysts for hydrogen evolution reaction.

## 1. Introduction

With the energy crisis and environment pollution becoming serious, hydrogen energy has attracted wide attention around the world because of its renewability and pollution-free combustion products [1,2]. Among various hydrogen production methods, water electrolysis is viewed as the most promising and economical route due to its abundant source [3], in which a highly active electrocatalyst is the key to reducing overpotential for achieving efficient hydrogen production. Especially, an electrocatalyst with high activity over a wide pH range is crucial for practical applications [4]. However, in acidic and alkaline electrolytes, the hydrogen evolution reaction (HER) in water electrolysis involves different processes and forms different intermediates. Therefore, there are relatively few electrocatalysts that have good HER performance in both acidic and alkaline environments [4,5]. Up to now, among various HER catalysts, Pt and its derivates still are the best commercial catalysts because of their high activity and good stability. But high cost and scarce resources limit their large-scale application [6,7].

MoP as a transition-metal phosphide has Pt-like electronic structure, high conductivity, and corrosion resistance properties [8,9] and therefore presents potential value for hydrogen production in acid and alkaline environments. But even so, the catalytic activity of the pristine MoP is far from the demands of practical application and needs to be further improved [10,11]. Meanwhile, Wang et al. found that the P atoms in MoP have a near-zero Gibbs free energy of hydrogen adsorption, which means that these P atoms create a large number of edge active sites for the hydrogen evolution reaction. Ultimately, compared with Mo and Mo_3_P, with a lower degree of phosphorization, highly phosphorized MoP exhibits higher activity in the hydrogen evolution reaction [12]. Against this background, to achieve a uniform and ideal phosphorization result, some soluble phosphates (including (NH_4_)_2_HPO_4_, NaH_2_PO_2_, etc.) have been widely used to prepare MoP and MoP-based electrocatalysts [6,13,14]. For example, CoP/MoP@NPC synthesized using (NH_4_)_2_HPO_4_ as a precursor via a one-step phosphating process showed a low HER overpotential of 183 mV at 10 mA/cm^2^ in 0.5 M H_2_SO_4_ [6]. The Ru-MoP NWAs/CFP prepared by phosphorization using NaH_2_PO_2_ as the phosphorus source exhibited remarkable catalytic performance for the HER over a wide pH range [13]. However, the widespread use of soluble phosphates not only contributes to water eutrophication, which reduces dissolved oxygen levels and disrupts ecological balance, but also raises the synthesis cost of MoP. Based on the above reasons, exploring green and low-cost preparation methods of MoP-based electrocatalysts with high catalytic activity is the key to realizing the application of MoP-based catalysts in the field of electrocatalytic hydrogen production.

In this work, P powder was used as the phosphorus source to synthesize MoP nanocrystal via a straightforward and cost-efficient high-temperature phosphidation approach. In contrast to soluble salts like (NH_4_)_2_HPO_4_ and NaH_2_PO_2_, P powder is water-insoluble and more economically viable. Further, using the as-prepared MoP as a reactant to synthesize Ru/MoP composite exhibited comparable or superior HER catalytic activity in both acidic and alkaline solutions compared to other previously reported excellent Ru- or MoP-based catalysts. The experimental results confirmed that the higher catalytic activity of Ru/MoP compared with pristine Ru and MoP is due to the presence of more active sites on the surface.

## 2. Experimental Section

### 2.1. Reagents

Molybdenum trioxide (99.9%), commercial Pt/C catalyst (20% Pt), phosphorus standard (98.5%), and polyvinylpyrrolidone (PVP, mean molecular weight of 58,000) were bought from Macklin; ethyl alcohol (AR, 99.7%) and ruthenium chloride hydrate (RuCl_3_·*x*H_2_O, 97%) were obtained from Innochem; Nafion solution (5%) was purchased from Adamas. All reagents were directly used as received without further purification.

### 2.2. Preparation of MoP, Ru, and Ru/MoP

**Figure 1:**
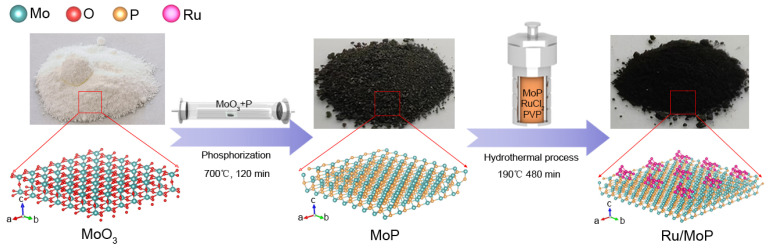
Schematic illustration of the synthetic process of MoP and Ru/MoP electrocatalysts.

The fabrication procedure of MoP and Ru/MoP nanocrystals is schematically illustrated in Figure 1. Firstly, the MoP was prepared via a high-temperature phosphorization method [15]. The mixture of MoO_3_ and P powder with a mass of 1:1 was placed in a quartz boat. The quartz boat was put in a small quartz tube (25 mm diameter, 100 cm long). Then, the small quartz tube was inserted into a big quartz tube (50 mm diameter, 180 cm long) mounted inside a horizontal tube furnace, ensuring that the quartz boat was kept away from the center of the tube furnace. Before heating, the big quartz tube was evacuated to remove the air inside the tube. Subsequently, the tube furnace was heated to 700 °C at 27 °C/min, and then, the quartz boat was quickly pushed to the center of tube furnace, which was then maintained at 700 °C for 120 min under 200 SCCM (standard cubic centimeter per minute) H_2_ flow. After the growth process, the black product formed in the quartz boat was MoP.

For the preparation of Ru/MoP, the fabrication process mainly contained two steps. Typically, the as-grown MoP (0.127 g) and RuCl_3_·*x*H_2_O with molar ratio of 1:1 was dissolved in 20 mL deionized water, then 0.05 g PVP was added under continuous stirring. Afterward, the mixture was introduced into a 40 mL stainless steel reactor, which was placed in an oven at 190 °C and maintained for 480 min. The obtained product was washed three times using ethanol, acetone, and deionized water, respectively, and then dried in an oven at 60 °C. Secondly, the collected black power was annealed at 500 °C under 200 SCCM H_2_ flow for 120 min. Finally, the Ru/MoP was obtained. For the synthesis of Ru, the experimental setup and procedure were the same as those of Ru/MoP preparation; the only difference was the absence of MoP in the reactants.

### 2.3. Characterization

The crystal structure of the sample was characterized using an X-ray diffractometer (XRD, Ultima IV, Rigaku, Japan) with Cu Kα radiation (λ = 1.5406 Å) at a scanning rate of 5° min⁻^1^ in the 2θ range of 10° to 90°. Raman spectra were collected using a confocal Raman microspectrometer (HORIBA LabRAM Odyssey, HORIBA, Kyoto, Japan) with a 532 nm laser excitation source. The morphology and surface features of the samples were observed using field-emission scanning electron microscopy (FE-SEM, Quanta 250 FEG, FEI, Quanta, Ann Arbor, MI, USA) operated at an accelerating voltage of 5 kV. The chemical states and elemental composition of the samples were analyzed by X-ray photoelectron spectroscopy (XPS, ThermoFisher ESCALAB 250Xi, Thermo Fisher Scientfic, Waltham, MA, USA) with Al Kα radiation (1486.6 eV), and the binding energies were calibrated using the C 1s peak at 284.8 eV as a reference. The microstructure and chemical composition of the samples were further investigated using high-resolution transmission electron microscopy (HR-TEM, Thermo Scientific TF-G20, USA) equipped with energy-dispersive X-ray spectroscopy (EDS), operated at an accelerating voltage of 200 kV.

### 2.4. Electrochemical Measurements

The electrochemical measurements were implemented at 25 °C using a three-electrode system connected to an electrochemical workstation (ZAHNER-Thales) equipped with Thales XT 5.2.0 software (as shown in Figure S1). The catalyst suspension was prepared by dispersing the as-prepared sample (4 mg) into 1 mL of water/ethanol mixture (*v*/*v* = 4:1) containing 80 μL Nafion solution and sonicating for 3 min. Subsequently, a total of 20 μL suspension was loaded onto a glass carbon (GC) electrode (3 mm diameter) and naturally dried at room temperature. Meanwhile, for evaluating the HER performance of the as-prepared catalyst, the commercial Pt/C catalyst of the same mass was loaded onto the GC electrode. A graphite rod was used as the counter electrode, and an Ag/AgCl electrode and a standard Hg/HgO electrode were selected as reference electrodes in 0.5 M H_2_SO_4_ and 1.0 M KOH, respectively. In the process of measurement, the linear sweep voltammetry (LSV) was conducted at a scan rate of 10 mV·s^−1^. Meanwhile, all polarization curves were iR-corrected through the “ohmic drop” function of the instrument. A cyclic voltammetry (CV) test was performed at diverse scan rates of 20, 40, 60, 80, and 100 mV·s^−2^, respectively.

## 3. Results and Discussion

**Figure 2:**
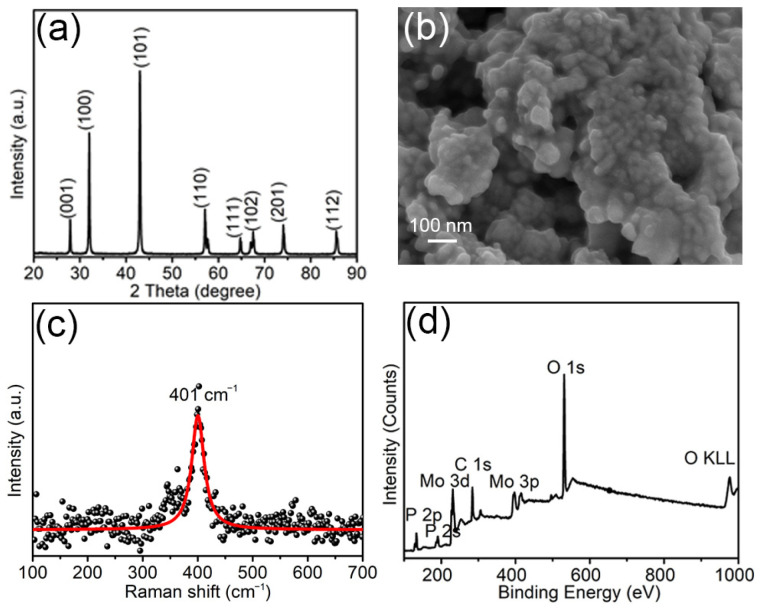
Characterization on converted MoP: (**a**) XRD pattern, (**b**) SEM image, (**c**) Raman spectrum, and (**d**) XPS survey spectra.

The crystal structure of the as-synthesized sample via high-temperature phosphorized MoO_3_ is firstly characterized. Figure 2a gives the corresponding XRD pattern. The obvious diffraction peaks at 27.9°, 32.0°, 43.0°, 57.1°, 64.8°, 67.5°, 74.1°, and 85.6° can be observed, which match well with the (001), (100), (101), (110), (111), (102), (201), and (112) crystal planes of hexagonal MoP (JCPDS NO. 65.6487). Besides these, no additional peaks are observed. Figure 2b shows the SEM image of the as-synthesized MoP at 50,000× magnification under 5.0 kV acceleration voltages. It can be seen that the MoP comprises particles with a size of tens of nanometers. The Raman spectrum of the as-synthesized MoP is exhibited in Figure 2c. The only visible peak appears at 401 cm^−1^, which is consistent with reported MoP in the literature [16,17]. Figure 2d presents the XPS survey spectrum of the as-synthesized MoP, in which the P 2p, P 2s, Mo 3d, and Mo 3p peaks from MoP are clearly visible, while the C 1s peak is ascribed to the adventitious carbon. At the same time, the presence of O 1s and O KLL peaks in the sample means that it is inevitability oxidized in the atmosphere. Based on the above results, it can be inferred that the MoO_3_ has almost completely converted into MoP upon the high-temperature treatment.

**Figure 3:**
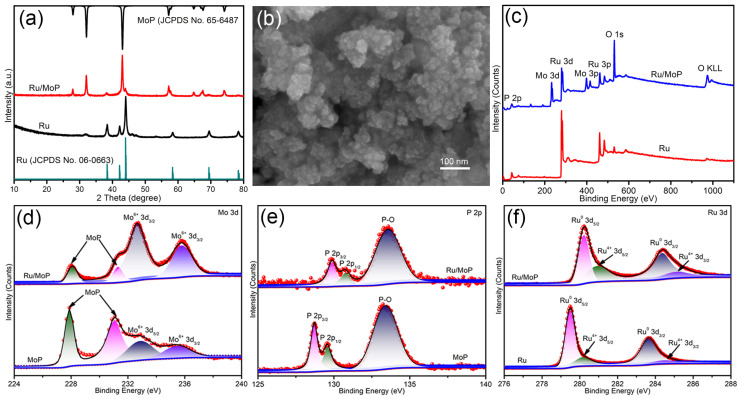
(**a**) XRD patterns of Ru/MoP and Ru. (**b**) SEM image of Ru/MoP. (**c**) XPS survey spectra of Ru/MoP and Ru. (**d**–**f**) Corresponding high-resolution XPS spectra of Mo 3d, P 2p, and Ru 3d.

Then, the converted MoP and RuCl_3_·*x*H_2_O were used as reactants to synthesize Ru/MoP composite by hydrothermal method. The corresponding XRD pattern of the as-synthesized sample is shown in Figure 3a. Besides the characteristic peaks of hexagonal MoP, the diffraction peaks of hexagonal Ru (JCPDS NO. 06-0663) are observed, indicating the coexistence of Ru and MoP phases in the sample. For the sample synthesized from only RuCl_3_·*x*H_2_O, the corresponding XRD pattern only contains the diffraction peaks of the hexagonal Ru, indicating the pure Ru. Figure 3b presents the SEM image of Ru/MoP, which reveals that the Ru/MoP comprises a large number of grains. However, compared with the pristine MoP, the particle size of Ru/MoP is much smaller, ranging from a few nanometers to over ten nanometers, which proves that the MoP is broken during the hydrothermal reaction. The XPS survey spectra of Ru and Ru/MoP are given in Figure 3c, from which the P 2p, Mo 3d, and Mo 3p peaks are observed in Ru/MoP, while the Ru 3d, Ru 3p, O 1s, and O KLL peaks are detected in Ru and Ru/MoP. The oxygen-related peaks show that both samples have different degrees of oxidation in the atmosphere. In order to investigate the chemical state of Mo in Mo and Ru/MoP, the high-resolution Mo 3d peaks were further analyzed. As shown in Figure 3d, the Mo 3d spectra can be divided into four peaks. The peaks at 227.9/228.1 eV and 231.1/231.3 eV are associated with MoP [10,18]. The peaks at 232.7/232.9 eV and 235.5/235.7 eV are assigned to Mo^6+^ [19,20]. This can be explained by the oxidation of MoP in air, where Mo atoms are oxidized from a lower valence state to Mo⁶⁺. Figure 3e gives the high-resolution XPS spectra of P, where a shoulder-like peak and a broad peak are observed in both MoP and Ru/MoP. The shoulder peak between 128 eV and 131 eV can be fitted into two bands, located at 128.7/129.8 eV and 129.6/130.7 eV, respectively, corresponding to P 2p_3/2_ and P 2p_1/2_ of the P−Mo bonds [21,22]. Notably, the P 2p peak exhibits a significant blueshift of 1.1 eV after the combination of MoP and Ru, while the broad peak at 133.5 eV, corresponding to P_2_O_5_ species [10,18], remains almost unchanged, indicating a strong electronic interaction between the MoP and Ru [23]. The Ru 3d peaks are shown in Figure 3f, which can be fitted to two couple peaks. The peaks at 279.5/280.2 eV and 283.7/284.4 eV are assigned to the 3d_5/2_ and 3d_3/2_ of metal Ru [24]. The other peaks located at 280.2/280.9 eV and 284.5/285.2 eV are ascribed to the Ru^4+^ 3d_5/2_ and 3d_3/2_ [24]. Compared with bare Ru, the peaks of Ru 3d in Ru/MoP shift to high energy, which is consistent with the blueshift of the P 2p peak, signifying the electrons transfer from Ru to MoP at the Ru/MoP interface.

**Figure 4:**
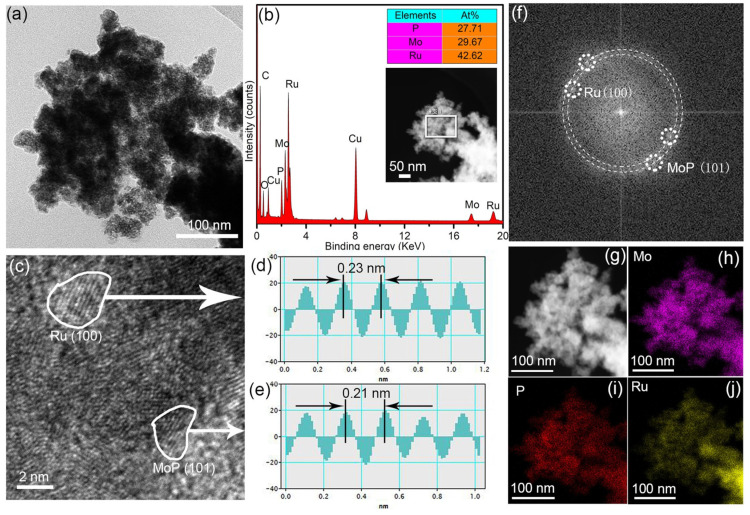
(**a**) Low-magnification TEM image of Ru/MoP. (**b**) TEM-EDS spectral image of Ru/MoP; insets are the element ratio and corresponding acquisition area for spectrum, respectively. (**c**) HRTEM image of Ru/MoP. (**d**,**e**) The corresponding inverse FFT images for the white circle region in (**c**). (**f**) FFT image of panel c. (**g**–**j**) High-angle annular dark field image of Ru/MoP and corresponding elemental mapping for Mo, P, and Ru.

Figure 4a displays the low-magnification TEM image of Ru/MoP, which is consistent with the above SEM result, where obvious particle structures are observed. The in situ EDS spectrum (Figure 4b) reveals that these nanoparticles are mainly composed of three considerable elements, P, Mo, and Ru, respectively (C and Cu signals come from the micro grid used for TEM test, while O is from the slight oxide of sample), and the atomic ratio of f P to Mo is close to 1:1, which further reveals that the composite has an Ru/MoP structure. Figure 4c is the high-magnification TEM (HRTEM) image. Multiple lattice fringes in different directions are observed. Corresponding interplanar distances of 0.34 nm and 0.21 nm were measured and are shown in Figure 4d,e, which are in agreement with those of the hexagonal Ru (100) and hexagonal MoP (101) planes [25,26], respectively. The corresponding fast Fourier transform pattern extracted from the HRTEM image is shown in Figure 4f. The diffraction rings represent the polycrystalline structure of Ru/MoP. By measuring the rings’ radii, it can be inferred that the large and small diffraction rings correspond to the (101) crystal plane of hexagonal MoP and (100) crystal plane of hexagonal Ru, respectively. Figure 4h–j present the results of element mapping of the Ru/MoP shown in Figure 4g. It can be seen that the Mo, P, and Ru are uniformly distributed over the whole sample, indicating the sample is homogeneous Ru/MoP composite.

**Figure 5:**
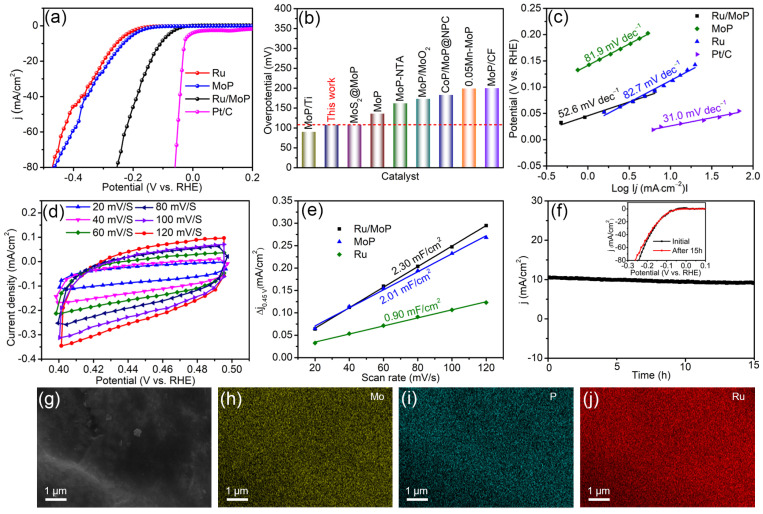
(**a**) LSV polarization cures of Ru, MoP, Ru/MoP, and Pt/C in 0.5 M H_2_SO_4_. (**b**) Comparison of overpotentials at 10 mA·cm^−2^ in 0.5 M H_2_SO_4_ between Ru/MoP and some previously reported MoP-based electrocatalysts. (**c**) Tafel slopes of Ru, MoP, Ru/MoP, and Pt/C for HER in 0.5 M H_2_SO_4_. (**d**) Typical CV curves of Ru/MoP. (**e**) Double-layer capacitance of Ru, MoP, and Ru/MoP. (**f**) Chronoamperometry curve of Ru/MoP for HER. The inset is the LSV curves of the initial Ru/MoP and after 15 h. (**g**–**j**) EDS mapping images of Ru/MoP electrocatalyst after stability testing in 0.5 M H_2_SO_4_.

Next, the electrocatalytic HER performance of the as-prepared samples was investigated. Figure 5a first gives the LSV curves of MoP, Ru, Ru/MoP, and commercial Pt/C in 0.5 M H_2_SO_4_. Undoubtedly, Pt/C has the best electrocatalytic activity, with an onset potential of near zero and an overpotential of 27 mV at the current density of 10 mA·cm^−2^. For the self-prepared electrocatalysts, the onset potential values of Ru, MoP, and Ru/MoP are 142.6, 132.6, and 31 mV, respectively, while the overpotential values at 10 mA·cm^−2^ (*η*_10_) are 258, 234, and 108 mV, respectively. As expected, the Ru/MoP composite has the highest electrocatalytic activity for HER in 0.5 M H_2_SO_4_. Although the catalytic activity of the as-synthesized Ru/MoP still needs to be improved compared to commercial Pt/C catalysts, it is impressive that the Ru/MoP shows better HER performance than previously reported MoP-based catalysts (as shown in Figure 5b and Table S1) in an acid environment, such as MoP/Ti (90 mV) [27], MoS_2_@MoP (108 mV) [28], MoP (136 mV) [12], etc. [6,29,30,31,32]. Furthermore, the corresponding Tafel slopes were obtained based on the LSV curve of the electrocatalysts, as shown in Figure 5c. The Tafel slope of Ru/MoP is 52.6 mV·dec^−1^, which is the smaller than those of bare MoP (81.9 mV·dec^−1^) and Ru (82.7 mV·dec^−1^). Generally speaking, a smaller Tafel slope means a faster reaction rate and better catalytic activity. Therefore, the Ru/MoP has the best hydrogen production activity compared with both of Ru and MoP, and it follows the Volmer–Heyrovsky reaction mechanism for HER. In order to elucidate the electrochemical active surface area (ECSA), the double-layer capacitances (C_dl_) of MoP, Ru, and Ru/MoP were calculated based on the CV curves (Figure 5d and Figures S2 and S3) measured at diverse scan rates in the potential range of 0.4−0.5 V (V vs. RHE), as shown in Figure 5e. The C_dl_ value of Ru/MoP is 2.3 mF/cm^2^, which is higher than those of single MoP (2.01 mF/cm^2^) and Ru (0.9 mF/cm^2^), proving that the synergistic effect between Ru and MoP can significantly increase the number of active sites [33]. In addition to electrocatalytic activity, stability is another important parameter for evaluating electrocatalytic performance. Figure 5f exhibits the chronoamperometry curves at an initial current density of 10 mA·cm^−2^. After 15 h, the current density of Ru/MoP hardly decreases. Furthermore, the elemental mapping images in Figure 5g–j reveal that Mo, P, and Ru elements still exist and are uniformly distributed throughout the sample after the stability test. The corresponding atomic ratios are shown in Table S3, where the atomic ratio of Mo to P is close to the stoichiometric ratio of 1:1 for MoP. Meanwhile, the atomic ratio of Ru is 50.62%, which is close to the Ru atomic ratio of 42.62% before the stability test of the Ru/MoP (as shown in Figure 4b). These results indicate that the Ru/MoP catalyst possesses outstanding stability for the hydrogen evolution reaction in acidic media.

**Figure 6:**
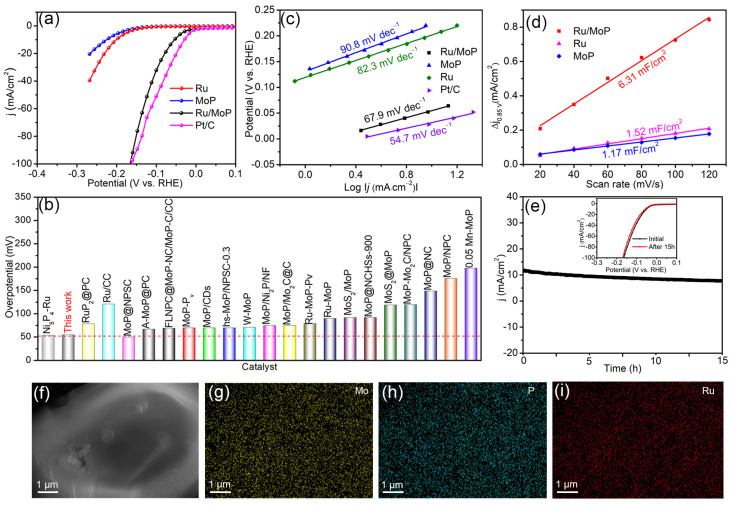
(**a**) LSV polarization cures of Ru, MoP, Ru/MoP, and Pt/C in 1.0 M KOH solution. (**b**) Comparison of overpotentials at 10 mA·cm^−2^ in 0.5 1.0 M KOH between Ru/MoP and previously reported Ru- and MoP-based electrocatalysts. (**c**) Tafel slopes of Ru, MoP, Ru/MoP, and Pt/C for HER in 1.0 M KOH. (**d**) Double-layer capacitance of Ru, MoP, and Ru/MoP. (**e**) Chronoamperometry curve of Ru/MoP for HER. The inset is the LSV curves of the initial Ru/MoP and after 15 h. (**f**–**i**) EDS mapping images of Ru/MoP electrocatalyst after stability testing in 1.0 M KOH.

According to previous reports, due to the slower kinetics of water dissociation in alkaline media than in acidic media [34], the catalytic activity of catalysts in an alkaline environment is usually much less than that in acidic environment [35,36]. In view of the good HER activity of Ru/MoP electrocatalyst in acid solution, its electrocatalytic activity in alkaline solution attracts our interest. Figure 6a shows the LSV curves of Ru/MoP, bare Ru, and pristine MoP catalysts in 1.0 M KOH solution. For Ru/MoP, the onset potential of near zero is almost identical to that of the Pt/C catalyst but significantly lower compared with Ru (124 mV) and MoP (136 mV). Meanwhile, the overpotential value of Ru/MoP is 54 mV at 10 mA·cm^−2^, approaching that of Pt/C (30 mV), far lower than those of Ru (199 mV) and MoP (224 mV). Above results indicate that the Ru/MoP has a higher HER activity than the pristine Ru and MoP and is also comparable or superior to other Ru- or MoP-based catalysts reported previously (see Figure 6b and Table S2), for example, Ni_5_P_4_-Ru [37], Ru/CC [38], Ru-MoP-Pv [14], MoP-Mo_2_C/NPC [39], etc. [11,14,26,28,31,34,39,40,41,42,43,44,45,46,47,48,49]. Figure 6c exhibits the Tafel plots of Pt/C, Ru, MoP, and Ru/MoP catalysts, and the corresponding Tafel slopes are 54.7, 82.3, 90.8, and 67.9 mV·dec^−1^, respectively, indicating all electrocatalysts undergo the Volmer–Heyrovsky mechanism during the HER in 1.0 M KOH solution. At the same time, compared with Ru and MoP, the Ru/MoP has the smaller Tafel slope, implicating faster HER reaction kinetics. As a result, a larger current density can be achieved for Ru/MoP at the same potential. To evaluate the ECSA of the self-prepared catalysts in alkaline solution, the CV curves of MoP, Ru, and Ru/MoP were measured and are shown in Figures S4–S6. Based on the tested results, corresponding C_dl_ values are calculated, as shown in Figure 6d. The C_dl_ values of MoP, Ru, and Ru/MoP are 1.17, 1.52, and 6.31 mF/cm^2^, respectively. Similar to that in acidic solution, the maximum C_dl_ value of Ru/MoP means that the combination of Ru and MoP can also provide more active sites for an alkaline water-splitting reaction [50]. Finally, the stability of the electrocatalytic reaction in alkaline solution was investigated. The time-dependent current density curve of Ru/MoP is presented in Figure 6e. It can be seen that the 10 mA·cm^−2^ current density is barely lower after 15 h. Meanwhile, Figure 6f–i display the elemental mapping images of the catalyst subsequent to the stability test. Evidently, the Mo, P, and Ru elements are still present within the sample and are evenly distributed. As illustrated in Table S4, the atomic percentages of P and Mo are 32.30% and 37.09%, respectively, closely matching the stoichiometric ratio of MoP. Furthermore, the Ru content remains largely unchanged compared to its initial atomic percentage before the catalytic stability test. These findings confirm that that the Ru/MoP catalyst demonstrates long-term stability during the alkaline HER reaction.

## 4. Conclusions

In this work, the MoP was prepared by a simple phosphorization route and used as one of the reactants to synthesize an Ru/MoP composite that shows excellent HER performance in both acidic and alkaline solutions. Compared with pristine Ru and MoP, the Ru/MoP has lower overpotentials at 10 mA·cm^−2^ and is comparable or superior to other Ru- or MoP-based catalysts reported previously. Besides this, the Ru/MoP composite also has lower onset potentials, smaller Tafel slopes, and more surface active sites. The present study thus provides a feasible route to design efficient MoP-based HER electrocatalysts.

## Data Availability

The original contributions presented in this study are included in the article/Supplementary Material. Further inquiries can be directed to the corresponding authors.

## Supplementary Materials

The following supporting information can be downloaded at: https://www.mdpi.com/article/10.3390/ma18051184/s1, Figure S1: Optical picture of the test setup; Figure S2: Cyclic voltammogram (CV) curves of MoP in 0.5 M H_2_SO_4_; Figure S3: Cyclic voltammogram (CV) curves of Ru in 0.5 M H_2_SO_4_; Figure S4: Cyclic voltammogram (CV) curves of MoP in 1.0 M KOH; Figure S5: Cyclic voltammogram (CV) curves of Ru in 1.0 M KOH; Figure S6: Cyclic voltammogram (CV) curves of Ru/MoP in 1.0 M KOH; Table S1: Comparison of HER performance for Ru/MoP with other previously reported MoP-based electrocatalysts in 0.5 M H_2_SO_4_ solution. Table S2: Comparison of HER performance for Ru/MoP with other previously reported Ru- and MoP-based electrocatalysts in 1.0 M KOH solution. Table S3: Element ratio of of Ru/MoP electrocatalyst after stability testing in 0.5 M H_2_SO_4_. Table S4: Element ratio of of Ru/MoP electrocatalyst after stability testing in 1.0 M KOH.
