# Supplementary material for: Facile Synthesis of MoP and Its Composite Structure with Ru as an Efficient Electrocatalyst for Hydrogen Evolution Reaction in Both Acidic and Alkaline Conditions
Source: Materials (Basel). 2025 Mar 6;18(5):1184. doi: 10.3390/ma18051184 (PMC11901484; doi:10.3390/ma18051184)
Supplement: Supplementary file 1 [file materials-18-01184-s001.zip › materials-3472416-supplementary.pdf]

Supplementary Material

Facile Synthesis of MoP and Its Composite Structure with Ru as an Efficient Electrocatalyst for Hydrogen Evolution Reaction in Both Acidic and Alkaline Conditions

Pinyun Ren [1,2,*], Rui Wang [1], Yujie Yang [1], Tianyu Wang [1], Yilun Hong [1], Yi Zheng [1], Qianying Zheng [1], Xianpei Ren [2], and Zhili Jia [3,*]

[1] School of Photoelectric Engineering, Changzhou Institute of Technology, Changzhou 213032, China

[2] College of Physics and Electronic Engineering, Material Corrosion and Protection Key Laboratory of Sichuan Province, Artificial Intelligence Key Laboratory of Sichuan Province, Sichuan University of Science and Engineering, Zigong 643000, China

[3] Center for Advanced Measurement Science, National Institute of Metrology, Beijing 100029, China

***** Correspondence: renpy@czu.cn (P.R.); jiazl@nim.ac.cn (Z.J.)

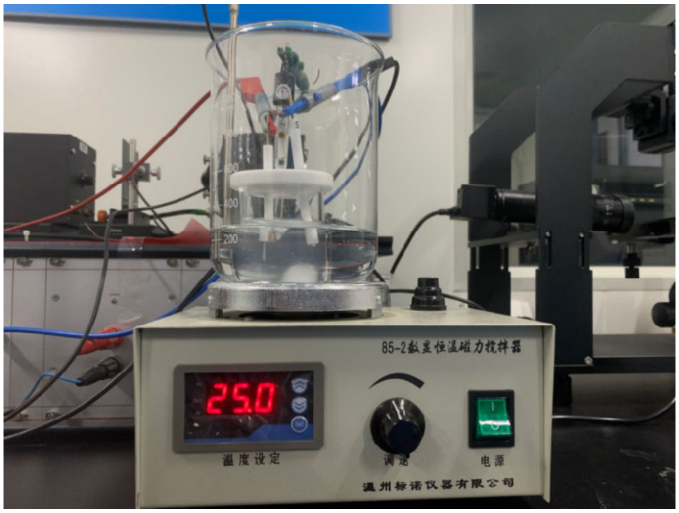

Figure S1. Optical picture of the test setup

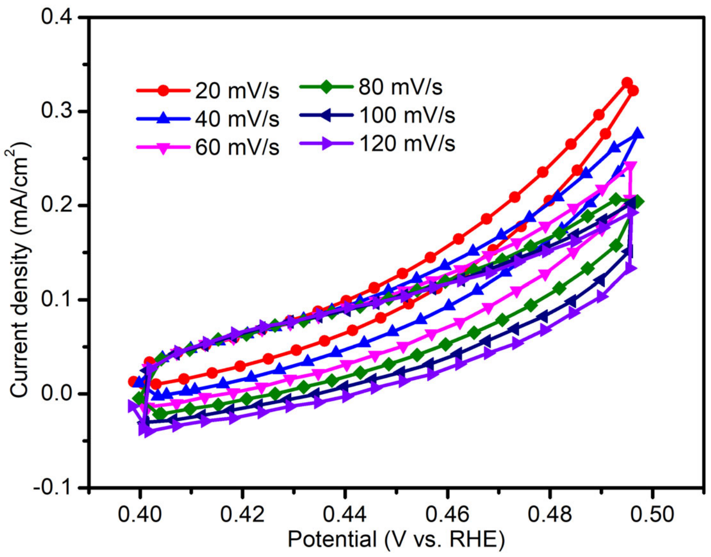

Figure S2. Cyclic voltammogram (CV) curves of MoP in 0.5 M H_2SO_4

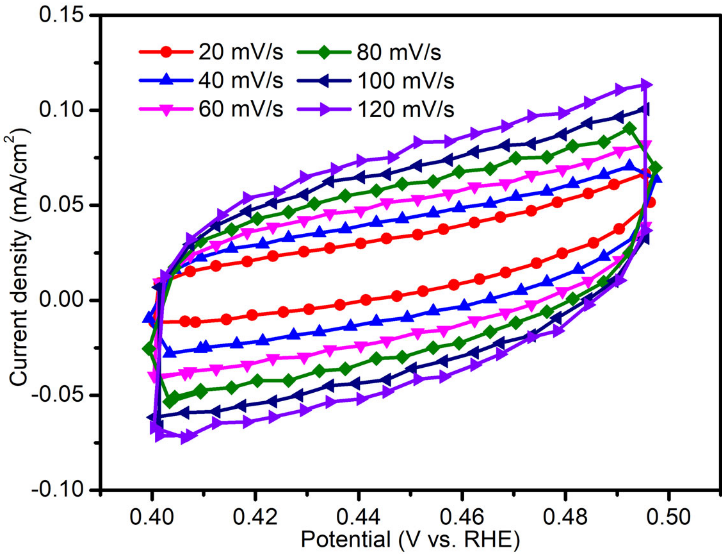

Figure S3. Cyclic voltammogram (CV) curves of Ru in 0.5 M H_2SO_4

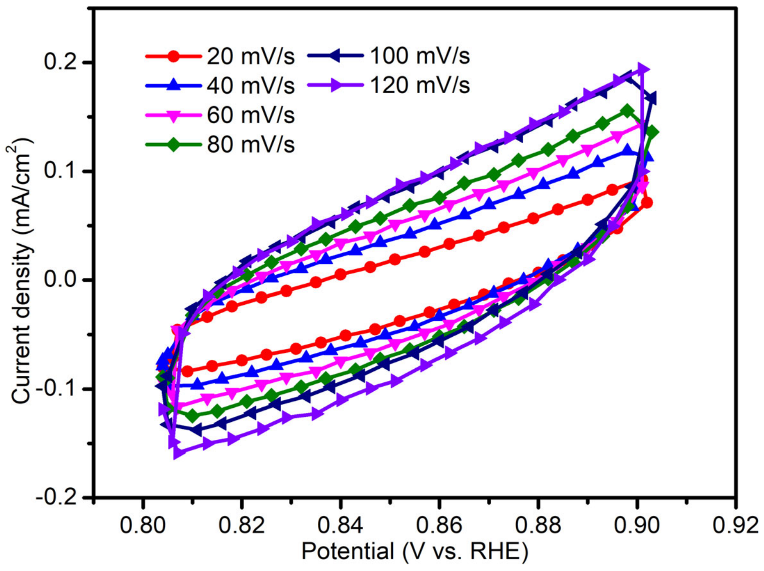

Figure S4. Cyclic voltammogram (CV) curves of MoP in 1.0 M KOH

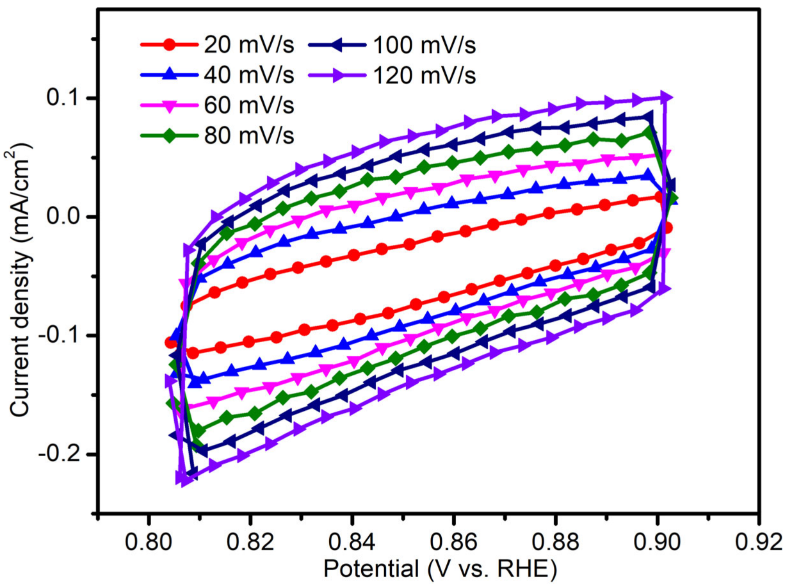

Figure S5. Cyclic voltammogram (CV) curves of Ru in 1.0 M KOH

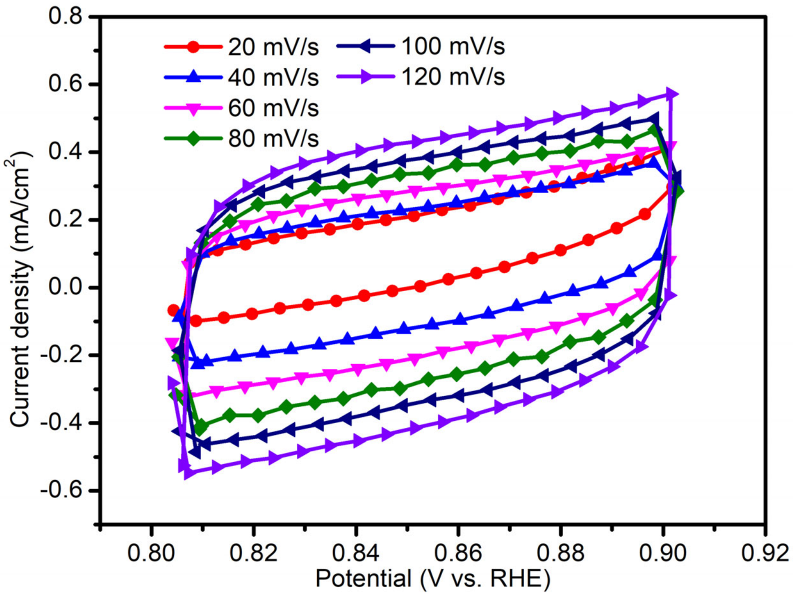

Figure S6. Cyclic voltammogram (CV) curves of Ru/MoP in 1.0 M KOH

Table S1. Comparison of HER performance for Ru/MoP with other previously reported MoP-based electrocatalysts in 0.5 M H_2SO_4 solution.

**Electrocatalyst**	**Overpotential (mV) @10 mA·cm^{-1}**	**References**
MoP/Ti	90	*Chem. Mater.*, 2014, 26: 4826
MoS_2@MoP	108	*Nanoscale*, 2016, 8: 11052
MoP	136	*Energy Environ. Sci.*, 2014, 7: 2624
MoP-NTA	162	*Int. J. Hydrogen Energy*, 2017, 42: 14566
MoP/MoO_2	173	*Mater. Lett.*, 2019, 241: 227
0.05 Mn-MoP	199	*Appl. Surf. Sci.*, 2021, 551: 149321
MoP/CF	200	*Appl. Catal. B Environ.*, 2015, 164: 144

Table S2. Comparison of HER performance for Ru/MoP with other previously reported Ru- and MoP-based electrocatalysts in 1.0 M KOH solution.

Electrocatalyst	Overpotential (mV) @10 mA·cm^{-1}	References
Ni_5P_4-Ru	54	*Adv. Mater.*, 2020, 32: 1906972
RuP_2@PC	78.9	*J. Mater. Chem. A*, 2021, 9: 12276
Ru/CC	121	*Appl. Catal. B Environ.*, 2019, 249: 91
MoP@NPSC	50	*ACS Appl. Mater. Interfaces*, 2020, 12: 49596
A-MoP@PC	67	*Nano-Micro Lett.*, 2021, 13: 215
FLNPC@MoP-NC/ MoP-C/CC	69	*Adv. Funct. Mater.*, 2018, 28: 1801527
MoP-Pv	70	*Nanoscale*, 2023, 15: 1357
MoP/CDs	70	*Nano Energy*, 2020, 72: 104730
hs-MoP/NPSC-0.3	70	*ChemSusChem*, 2019, 12: 4662
W-MoP	71	*ACS Appl. Nano Mater.* 2021, 4: 5992
MoP/Ni_2P/NF	75	*J. Mater. Chem. A*, 2017, 5: 15940
MoP/Mo_2C@C	75	*ACS Appl. Mater. Interfaces*, 2017, 9: 16270
Ru-MoP-Pv	79	*Inorg. Chem.*, 2023, 62: 9687
Ru-MoP	90	*Inorg. Chem.*, 2023, 62: 9687
MoS_2/MoP	92	*Small*, 2020, 16: 2002482
MoP@NCHSs-900	92	*Angew. Chem.*, 2020, 132: 9067

MoS_2@MoP	119	*Nanoscale*, 2016, 8: 11052
MoP-Mo_2C/NPC	120	*Chem. Eng. J.*, 2022, 431: 133719
MoP@NC	149	*Appl. Catal. B Environ.*, 2020, 263: 118358
MoP/NPC	176	*Chem. Eng. J.*, 2022, 431: 133719
0.05 Mn-MoP	198	*Appl. Surf. Sci.*, 2021, 551: 149321

Table S3. Element ratio of of Ru/MoP electrocatalyst after stability testing in 0.5 M H_2SO_4.

Elements	At%
P	23.23
Mo	26.15
Ru	50.62

Table S4. Element ratio of of Ru/MoP electrocatalyst after stability testing in 1.0 M KOH.

Elements	At%
P	32.30
Mo	37.09
Ru	30.61
